# Contamination Assessment and Temporal Evolution of Nitrates in the Shallow Aquifer of the Metauro River Plain (Adriatic Sea, Italy) after Remediation Actions

**DOI:** 10.3390/ijerph191912231

**Published:** 2022-09-27

**Authors:** Marco Taussi, Caterina Gozzi, Orlando Vaselli, Jacopo Cabassi, Matia Menichini, Marco Doveri, Marco Romei, Alfredo Ferretti, Alma Gambioli, Barbara Nisi

**Affiliations:** 1 Dipartimento di Scienze Pure e Applicate, Università degli Studi di Urbino Carlo Bo, Via Ca’ Le Suore 2/4, 61029 Urbino, Italy; 2 Dipartimento di Scienze della Terra, Università di Firenze, Via G. La Pira 4, 50121 Firenze, Italy; 3 CNR-IGG Istituto di Geoscienze e Georisorse, Consiglio Nazionale delle Ricerche, Via G. La Pira 4, 50121 Firenze, Italy; 4 CNR-IGG Istituto di Geoscienze e Georisorse, Consiglio Nazionale delle Ricerche, Via G. Moruzzi 1, 56124 Pisa, Italy; 5 ASET SpA, Via Luigi Einaudi 1, 61032 Fano, Italy

**Keywords:** nitrate pollution, nitrate time series analysis, groundwater, hydro-geochemistry

## Abstract

Over the last decades, groundwater resources at global level have suffered a significant deterioration due to nitrate pollution, mainly related to the input of agricultural fertilizers, manure, sewage, and untreated urban and industrial effluents. The most impacted waters are those forming surface and shallow reservoirs, which usually play a key role in supplying waters to civil, agricultural, and industrial activities. The terminal portion of the Metauro River plain, located in central Italy along the Adriatic Sea coastline, hosts a strategic phreatic aquifer that, along with the surface water of the Metauro River, supplies water to the local population (i.e., about 60,000 people). This shallow coastal aquifer experiences a long-lasting story of nitrate contamination since the 1970s when the increase in the use of agricultural fertilizers contributed to very high levels of pollution (NO_3_^−^ > 100 mg/L). This fact prompted the local authorities to carry out remediation actions that involve a pumping system to inject the NO_3_^−^-poor waters from the Metauro River course directly into the shallow aquifer. The present work was aimed at defining the contamination of nitrates in this important water resource. The main geochemical characteristics and the temporal evolution of NO_3_^−^ concentrations (between 2009 and 2020), in the shallow coastal aquifer of the Metauro River plain, were analyzed by means of classical geochemical analyses and multivariate methods accounting for the compositional nature of the data, to assess the efficiency of the in-situ remediation over time.

## 1. Introduction

Large volumes of water resources at the global level are seriously affected by nitrate contamination, mainly deriving from the large use of: (i) nitrogen fertilizer in agricultural practices; (ii) wastewater treatment; (iii) domestic waste; and (iv) industrial discharges [1,2,3,4,5,6,7]. As a result, the N-compounds can affect aquifers where the concentration of dissolved NO_3_^−^ may significantly increase. High contents of nitrate in drinking water may result in serious health problems related to methemoglobinemia, cancer, birth defects, and other adverse effects [8], while nitrate excess in surface waters may contribute to eutrophication [9]. The most impacted waters are surface bodies (lakes, rivers, dams) and shallow aquifers, the latter mostly hosted in alluvial plains where highly permeable deposits usually dominate (e.g., [10]). Globally, these aquifers represent about 50% of drinking water and approximately 40% of irrigation water [7,11]. About 70% of the population in the European countries relies on groundwater exploitation for human consumption [12]. Shallow to deep aquifers thus play a key role in sustaining civil, agricultural, and industrial activities. In Europe, nitrate pollution by diffuse sources was first targeted by the Nitrate Directive (91/676/CEE) and subsequently by the Water Framework Directive (WFD, hereafter) 2000/60/EC (*Establishing a framework for Community action in the field of water policy*, https://eur-lex.europa.eu/eli/dir/2000/60/oj (accessed on 2 February 2022)), which also defined “Vulnerable Nitrate Zones”, intended as territories that drain nitrate-polluted (or at risk of pollution) waters. They are identified as those containing, or that could be containing, >50 mg/L of NO_3_^−^, thus requiring geochemical characterization and/or implementation of action programs to reduce water pollution by N-bearing compounds. WFD was transposed in Italy by the Italian Law Decree 152/2006 (3 April 2006, and subsequent amendments) that governs the individuation and characterization of the water bodies in terms of preservation, regulation, management, and remediation. Despite these growing efforts to reduce NO_3_^−^ inputs at global and local levels, nitrate is still one of the most widespread pollutants in groundwater (e.g., [2,3,5,7,13,14]) and a major concern due to its high solubility in water and difficultly to be fixed in the soil [15]. Moreover, defining the sources of NO_3_^−^ to groundwater is often challenging, especially where multiple (anthropogenic and natural) NO_3_^−^ sources are present in the aquifer systems [16]. Remediation actions to reduce nitrate concentration in groundwater systems are commonly used, although geological and hydrogeochemical features, as well as the quality of reactive materials, may affect the efficiency of these systems [17].

**Figure 1:**
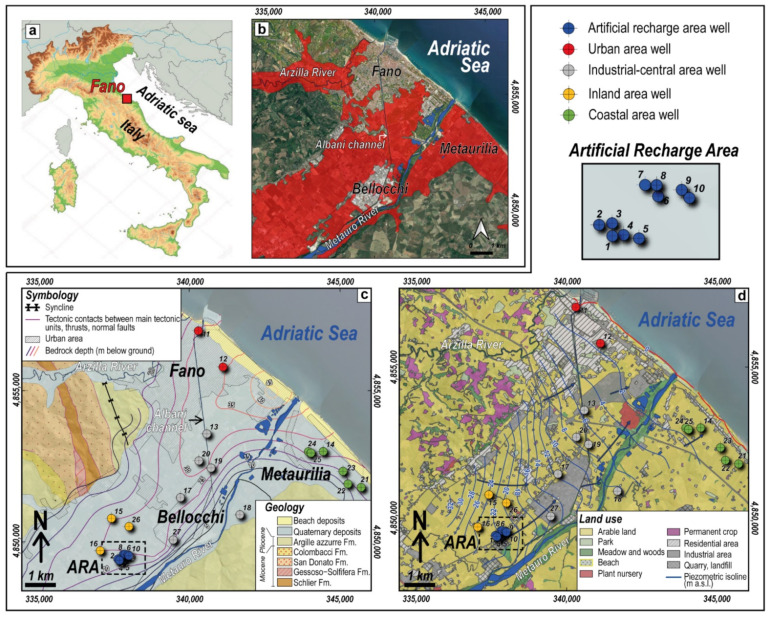
(**a**) General location and (**b**) map of the nitrate vulnerable zones (in red) of the study area (modified from Regione Marche [20]); (**c**) geological map (modified from Conti et al. [21]) in which the contour lines of bedrock depth are reported (after Taussi et al. [22]); (**d**) land use map (modified from Regione Marche [23]) in which the averaged piezometric isolines referred to 2019–2020 are represented (after Nisi et al. [18]). Reference system: WGS84-UTM 33N.

In this work, the shallow aquifer of the Metauro River plain, located in the northern coastal part of the Marche Region (Central Eastern Italy) and overlooking the Adriatic Sea (Figure 1a), was investigated since it was declared by the regional administration as highly vulnerable to nitrates (Figure 1b) due to the remarkably high NO_3_^−^ concentrations (up to >100 mg/L; [18]). The shallow aquifer is only a limited portion of the drinking water supplied by the local water management company (named ASET Ltd., Fano, Italy) to the local population (about 60,000 people living in the city of Fano), being the main volume provided by the surface water of the Metauro River. However, waters from the phreatic aquifer are of paramount importance since the extraction rates grow considerably in summertime (June to September). In this period, the hydrometric level of the Metauro River significantly drops, and agricultural practices and tourist activity increase the water demand, sometimes leading to sporadic water shortages and increasing costs of purification. Consequently, in the early 1990s the local water management company was forced to install a pumping system to inject the NO_3_^−^-poor waters (<10 mg/L; [18]) from the Metauro River directly into the aquifer (Artificial Recharge Area: ARA) to minimize as much as possible the impact due to the N-bearing species on drinking water [18]. In this study, the main geochemical characteristics of the shallow coastal aquifer of the Metauro River plain were investigated to highlight the extension of the NO_3_-polluted area and the relationships with the other physicochemical parameters using classical geochemical analyses, while multivariate methods were applied to account for the compositional nature of the data [19]. Eventually, the evolution and temporal trends of NO_3_^−^ concentrations between 2009 and 2020 were evaluated by using Median-Based Linear Regression Models to assess the efficiency of in-situ remediation over time.

## 2. Geology, Hydrogeology and Land Use

The study area is situated in the terminal part of the Metauro River catchment, where the valley of the river passes to a coastal plain down to the Adriatic coastline. The northernmost part of the area is also influenced by the Arzilla River, a torrential stream whose mouth is located in the northern boundary of the city of Fano (Figure 1b). The valley is bordered by outcropping Miocene-to-Pliocene formations, mainly composed by clays and silty clays, marlstones and, to a less extent, evaporites, which form the relatively impermeable bedrock above which the Quaternary alluvial-marine sediments were deposited (Figure 1c). The latter mostly consist of gravel, sand and silty-clay, with interbedded sand, clay and sandy-silty clay that locally prevail [18,22,24,25]. The sandy component becomes prevalent along the shoreline, where beach deposits dominate. The bedrock depth, as well as the Quaternary deposits thickness, varies widely along the plain ranging from <5 m up to ~50 m below the ground surface [22,26]. The substrate shows increasing depth from ~25 m below the ground, in the SW part of the study area, up to ~50 m near the mouth of the Metauro river (Figure 1c; [22,26]) where a well-developed coastal fan occurs [26,27]. The sedimentary infill and the substrate are shallower at the borders of the valley, where the bedrock approaches the surface and occasionally outcrops in the surrounding hills. In the coastal area of Metaurilia and near the river course, the bedrock shows marked slopes due to the proximity of the hills, deepening from <10 m up to ~30 m below the ground in a narrow space (i.e., <1.5 km; Figure 1c).

The permeable deposits of the Alluvial Plain (AP) favored the development of an unconfined and generally single-layer aquifer system, whose maximum thickness can reach ~40 m [18,22,24,25]. However, the sporadic presence of silty-clay layers in the dominant gravelly and sandy deposits produces local multilayer arrangements. The reconstruction of the piezometric levels indicate a SW to NE circulation and vary from ~32 to <1 m a.s.l. approaching the coastline (Figure 1d; [18]). The Metauro River is apparently draining the aquifer system, although the river/aquifer water level relationship varies according to the hydrological regime. The saturation level of the aquifer varies from ~1 m up to ~20 m below the ground [18,22], with seasonal variations up to ± 1 m [18]. The minimum levels of the saturation depth are found near the coastline and along the Metauro river course, while NW of ARA the maximum ones are recorded (Figure 1d). The annual rainfall ranges between 700 and 780 mm [18,28], mainly occurring in the wet period (October–May), while the mean annual temperature is ~14.6 °C (reference period 2009–2019; [22]).

The land use is almost entirely devoted to arable fields and urban (residential and industrial) settlements (Figure 1d; [23]). Some permanent crops (e.g., vineyards, olive groves) are present in the surrounding hills, while meadows and woods are mostly located along, or near, the river course. Other main anthropogenic activities in the study area are related to gravel quarries, plant nurseries, and industrial plants mainly associated with metallurgy, shipbuilding, paints, glasses, manufacturing of Fe- and Al-alloys.

## 3. Materials and Methods

### 3.1. Geochemical Methods

The chemical data used in this study were made available by the local water supply company (ASET Ltd.) in the framework of the bilateral Interreg Italy-Croatia project ASTERIS: Adaptation to Saltwater intrusion in sEa level RIse Scenarios (https://asteris.uniurb.it/ (accessed on 5 February 2022)). To ensure that the water supplied corresponds to the current standards imposed by the national and local regulations, ASET personnel continuously analyze drinking water samples collected from the various wells located throughout the study area. The monitoring network consists of 27 water wells localized in the coastal part of the Metauro River plain (Figure 1c,d) from which, at least once per year, water chemistry is analyzed. Sixteen out of 27 wells are in the AP and 11 in the ARA, where the Metauro river waters are injected into the phreatic aquifer. The chemical parameters were not homogeneously analyzed during the different surveys. Nevertheless, pH, electrical conductivity, NO_3_^−^, Cl^−^, SO_4_^2−^, Na^+^, K^+^, Ca^2+^, Mg^2+^, are almost constantly determined along with some trace elements whose selection is variable among the different surveys. Both the main cations (Ca^2+^, Mg^2+^, Na^+^, and K^+^) and anions (Cl^−^, SO_4_^2−^ and NO_3_^−^) were sampled in PE bottles and analyzed within 24 h from sampling by ion chromatography at ASET Ltd. laboratory in Fano (Italy) using an 881 Compact IC-PRO Metrohm. The analytical error for the main ions was <5%. Carbonate species are not always measured since these analytes are not included in the WFD protocol. However, since 2015 HCO_3_^−^ has been added by ASET Ltd. to the analytical routine. When analyzed, HCO_3_^−^ was determined within 24 h from sampling by acidimetric titration; the titrating solution was 0.01 N HCl. If the whole water chemistry (HCO_3_^−^, Cl^−^, SO_4_^2−^, NO_3_^−^, Na^+^, K^+^, Ca^2+^, Mg^2+^) was available, the ionic balance was tested by means of the electroneutrality parameter [29]. If the results of the ionic charge balance error were exceeding ±10%, they were not considered in the further data processing. Besides the complete geochemical annual campaigns, 7 up to 14 annual surveys are carried out by ASET Ltd. to monitor NO_3_^−^ concentrations in the groundwater, according to the current national and regional legislation.

### 3.2. Compositional Data Analysis

If data are expressed as parts of a numerical total, such as mg/L in water chemistry, then they are compositional data (e.g., [30]). Typically, these data are vectors of always-positive values that quantify the contribution of the D parts of the entire composition, which represents the known total. Therefore, compositional data only provide relative information and are not free to vary independently, but they pertain to a constrained sample space with D-1 dimensions [19]. This peculiar space, named simplex, is governed by the Aitchison geometry that is based on perturbation and powering operations for moving points within the confined sample space [31]. Because of these properties, applying standard statistical methods designed for data belonging to the real Euclidean space to the statistical analysis of compositions may yield misleading results. This fact has been widely demonstrated by many applications in different research fields (e.g., [32,33]). This issue can be overcome when compositional data can be moved from the simplex to the usual real space by means of a set of transformations. The most common are isometric log-ratio (ilr) and centered log-ratio (clr) transformations [19,34]. The latter divides each D term of a composition by the geometric mean of all the considered parts, represented, in our case, by the chemical species. Accordingly, the obtained crl-coordinates bear the information interlinking each of the elements to the barycenter of the whole water composition [35], and therefore they should be interpreted in terms of relative variations. The transformed data can be correctly analyzed using traditional statistical methods, including multivariate techniques [36].

In this work, the physicochemical data were investigated by considering their compositional nature and using a robust compositional principal component analysis (PCA) as a comprehensive analytical method. The PCA was first applied to the entire groundwater dataset and then separately to the AP and ARA wells to further explore the dynamics of the two areas. The results were visualized using biplots that approximate the original data in a rank-two matrix through loadings and scores of the PCA, providing insights into data variability, correlations between variables and potential clustering [37]. Aitchison and Greenacre [38] adapted biplots for compositional data, and Filzmoser et al. [39] implemented a robust version of the compositional biplot based on clr-transformed data. The robust compositional PCA was performed using the R library “robCompositions” [40] in the open-source R environment [41]. Successively, the resulting scores and loadings were used as input factors within the R package “Factoextra” [42] to display the outputs [43]. Eventually, the results of the Compositional Data Analysis were compared and complemented with those obtained from the classical geochemical approach to gain a deeper understanding of the data.

### 3.3. Temporal Trend Analysis

A trend analysis using linear fitting was performed to investigate the evolution of nitrates during the considered time span, as well as their variability over time. Since our data were unevenly distributed with time, all the standard methods for time-series modelling and forecasting were excluded. First, the non-parametric Mann-Kendall trend test (MK test) was performed in R using the library “trend” [44] to determine whether monotonic trends exist in the experimental data [45,46]. The MK test is a non-parametric test, i.e., there is no underlying assumption about the normality of the data. The null hypothesis for the test is that no trend is present in the data. If the *p*-value is lower than the significance level of 0.01, then there is statistically significant evidence of an upward or downward trend in the considered data. Time trends were then estimated by using Median-Based Linear Regression Models (“mblm” library) [47], and more specifically, the Theil-Sen single median [48,49] and the Siegel repeated medians [50] methods. These approaches were preferred to the non-robust simple linear regression because they are insensitive to outliers and can be significantly more accurate for skewed and heteroscedastic data. The Theil-Sen median method computes the n (n − 1)/2 slopes of lines crossing all pairs of successive points along the x coordinate and then the intercepts of n lines having the calculated slopes. The medians of all the calculated slopes and intercepts are taken as slope and intercept estimators, respectively.

Siegel repeated medians compute, for each successive point, the n − 1 slopes of the lines connecting it to all the other values and calculate their medians. The median of the n obtained medians is then considered as slope estimator, a similar procedure being applied for the calculus of the intercept estimator. This approach results in more robust estimators compared to that of Theil-Sen method. Additionally, the LOWESS smoother, performing a robust locally weighted polynomial regression [51,52], was calculated using a smoother span of 0.6 and added to the plots of the original points.

One of the major concerns in linear regression analysis is the presence of residuals heteroscedasticity [53], which means that the variance of residuals is not stable but increases or decreases with fitted values of response variable (y). This implies that the obtained model is unable to explain some patterns in the response variable, which instead shows up in the residuals. This may result in an unreliable regression model, thus yielding wrong predictions. Therefore, it is important to check for heteroscedasticity by inspecting residuals vs. fitted values plot or alternatively using a test for heteroscedasticity of errors in regression. Breusch-Pagan test (BP test) against heteroscedasticity [54], included in the R library “lmtest”, was performed to check presence or absence of heteroscedasticity in the experimental data [55]. If the results of the test have a *p*-value lower than the significance level of 0.05, the null hypothesis that the variance of the residuals is constant can be rejected and be inferred that heteroscedasticity is present in the dataset.

## 4. Results

### 4.1. Hydrogeochemical Characterization

Table 1 shows the main summary statistics of the physicochemical data from the shallow coastal aquifer of the Metauro River plain. Nitrate has the highest number of available observations (1470), 926 belonging to ARA and 544 to AP, with only 1% of missing values (i.e., not analyzed), which were removed from the dataset before performing the time-trend analysis. Conversely, missing measurements are found in a significant percentage (up to >83%) for other variables, not allowing the application of replacement techniques. Silica, NO_2_^−^ and NH_4_^+^ have the lowest number of measurements and most of them were below the detection limit, thus they were excluded from data processing. About 16% (239) of all the analytical observations (1477, spanning from 2009 to 2020) correspond to complete analyses and are related to 2009, and 2015–2019. The main statistical parameters for each well are reported in the Supplementary Material.

The subset of complete analyses includes 118 observations from ARA and 120 from AP. Results of the ionic charge balance error reported that only 3 waters, belonging to AP, were not chemically balanced and thus unsuitable for classical geochemical elaboration. This has led to a total of 117 complete analyses considered for AP. Overall, 8 chemical variables and 3 physico-chemical parameters were considered (Table 2). Two missing values in the NO_3_^−^ data were imputed through a multiplicative non-parametric simple replacement method for missing data in compositional data sets [56,57]. The main statistical parameters of the balanced chemical analyses are reported in Table 2 for the two groups.

These parameters are also reported in Figure S1, where boxplots of the main physicochemical parameters (pH, electrical conductivity and Total Dissolved Solid) and main ions (HCO_3_^−^, Cl^−^, SO_4_^2−^, NO_3_^−^, Na^+^, K^+^, Ca^2+^, Mg^2+^) of the groundwater from the shallow aquifer are presented. In these diagrams, data of the balanced analyses are displayed, divided into ARA and AP samples. Waters exhibit pH values from 7.0 to 8.21, with ARA waters that show higher median values (7.6) with respect to those from AP (7.3). The TDS values span between 410 and 848 mg/L and from 510 and 1374 mg/L in ARA and AP, respectively. In general, the ARA samples vary in narrower ranges with respect to those from AP.

A similar behavior is also shown when the main ions are considered, since the ARA samples have lower Standard Deviation (SD) and Mean Absolute Deviation (MAD) than those of AP (Table 2). Median and mean values are not significantly different from each other, indicating the absence of relevant outlying observations in the dataset for both areas. Highly positively skewed distributions are detected for NO_3_^−^, SO_4_^2−^, Na^+^ and Ca^2+^ in ARA, and for Cl^−^, Na^+^ and K^+^ in AP (skewness > 1). Except for NO_3_^−^, SO_4_^2−^, Ca^2+^ in ARA and HCO_3_^−^, Na^+^ in AP, all data distributions are platykurtic (kurtosis < 3).

The main contribution in terms of TDS is given by HCO_3_^−^ (up to 388 and 573 mg/L for the ARA and AP samples, respectively) among the anions and by Ca^2+^ (up to 143 and 178 mg/L in ARA and AP, respectively) among the cations. Sulfate is generally present in higher concentrations (median values: ARA = 80.7 mg/L; AP = 107.4 mg/L) with respect to Cl^−^ (median values: ARA = 41.8 mg/L; AP = 82.0 mg/L).

Among the cations, setting aside Ca^2+^, Na^+^ is the second ion in terms of abundance, having a median value of 32.5 mg/L and 60.3 mg/L in the ARA and AP waters, respectively. Magnesium spans between 14 and 24 mg/L and from 14 and 45 mg/L with median values of 17.3 and 31.8 mg/L in ARA and AP, respectively. Potassium has the lowest contents (median values: ARA = 2.9 mg/L; AP = 3.6 mg/L).

**Figure 2:**
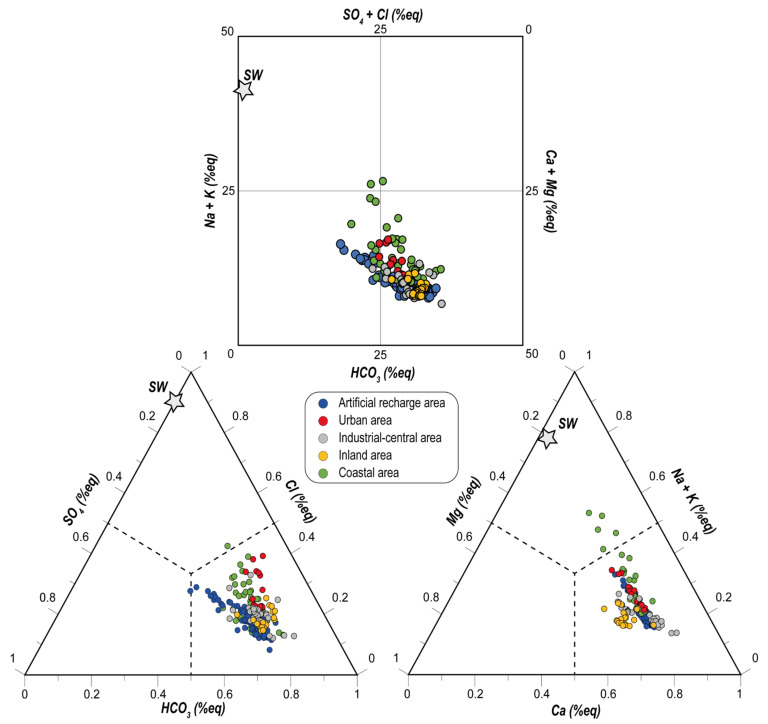
Langelier-Ludwig diagram and Cl-SO_4_-HCO_3_ and (Na+K)-Ca-Mg triangular diagram for the investigated samples. SW: seawater.

The waters chemistry of the shallow coastal area of the Metauro River plain can be classified through the square [58] and the Cl-SO_4_-HCO_3_ and (Na+K)-Ca-Mg triangular diagrams (Figure 2). In these plots, the well waters were divided according to their location. Waters from AP can thus be further subdivided into four different areas, as follows (Figure 1c,d): (i) urban, (ii) industrial-central, (iii) coastal, and (iv) inland. The square diagram shows that the main hydrogeochemical facies for all the waters is the bicarbonate alkaline-earth type. However, some samples pertaining to ARA are enriched in Cl+SO_4_ and Na+K, falling in the Ca-(Cl+SO_4_) field. In the same way, some samples from the coastal area show a marked increase in the Na+K contents, and a slight enrichment in Cl+SO_4_. The Cl-SO_4_-HCO_3_ ternary plot indicates that chloride and sulphate are always present in subordinate amounts, while the (Na+K)-Mg-Ca diagram displays enrichments in Na+K in almost all the water samples except for the inland ones. These increments are more evident in the waters sampled in the coastal area than in the others.

**Figure 3:**
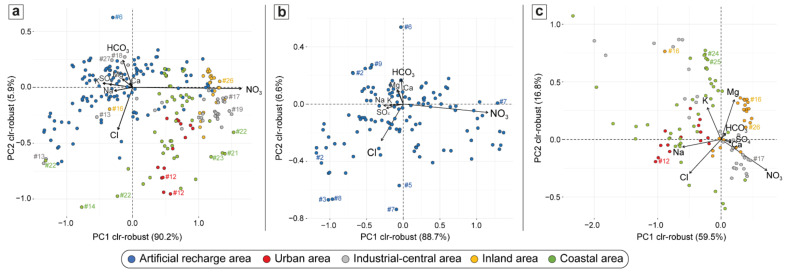
Robust compositional biplot of major species for: (**a**) all the investigated wells; (**b**) artificial recharge area (ARA) wells; and (**c**) alluvial plain (AP) wells. Scores are represented with different colors according to wells’ location. Variable labels in the plot represent the clr-transformed data of the corresponding species.

To characterize the component account of the monitored wells in accordance with the compositional nature of the data, clr-biplots were used. The results for the overall groundwater dataset are displayed in Figure 3a, while the separated analyses of ARA and AP wells are shown in Figure 3b and Figure 3c, respectively. The points are the PC scores, whereas black arrows represent the PC loadings from the origin (compositional barycenter). In the biplots with a good overall quality, the lengths of the arrows are proportional to the variability of each clr-variable, and attention is placed on the links between their vertices [59].

In the overall analysis (Figure 3a), the first component (PC1) accounts for 90% of the total variability, while the second one (PC2) explains 6%. The NO_3_^−^ log-ratio, with its long arrow, provides the greatest contribution to the first dimension on positive loadings, while SO_4_^2−^, K^+^, Na^+^ and the remaining variables oppose on negative loadings with significantly smaller contributions and a poorer quality of representation. In the second dimension, the major contributions are from Cl^−^ and HCO_3_^−^ log-ratios, on positive and negative loadings, respectively. Samples located in the industrial-central and inland areas are the most affected by NO_3_^−^ dominance (e.g., wells #17, #19 and #26) followed by those from the coastal and urban areas. Exceptions are found for a few measurements from water wells of the industrial-central (#13, #18, #27), inland (#16) and coastal (#22) areas. In contrast, the ARA scores appear less affected by nitrate contamination, even though some exceptions of a few samples are observed over the monitoring time. It is imperative to remember that dominance is here considered in a relative sense with respect to the compositional barycenter of the data. Waters from the urban and coastal areas show an increased contribution of Cl^−^ in comparison to the barycenter (e.g., #12, #14, #22). Accordingly, the NO_3_^−^ log-ratio, followed by the Cl^−^ and HCO_3_^−^ log-ratios, seem to significantly determine groundwater variability and indicate a significant compositional difference between ARA and AP wells.

This result is further confirmed by their separate analyses: PC1 and PC2 account for about 89% and 7% for ARA (Figure 3b), while 60% and 17% for AP (Figure 3c). In both cases, the variability is mostly described by NO_3_^−^ and Cl^−^ log-ratios. However, AP appears to have a more heterogeneous composition than ARA, with additional variables contributing to its overall variability (Figure 3c). In fact, a large contribution to the first dimension can also be attributed to the Na^+^ log-ratio, while on the second dimension Mg^2+^ and K^+^ log-ratios play a key role. Nitrate dominates in industrial-central and inland areas, whereas Cl^−^ and Na^+^ become significant in urban and coastal areas, confirming the previous results. A more relevant finding is the relative enrichment in Mg^2+^ and K^+^ for wells belonging to the coastal (e.g., #24 and #25) and inland areas (#16), which was not evident from either the comprehensive PCA analysis or the triangular diagrams.

### 4.2. Nitrate Contents

**Figure 4:**
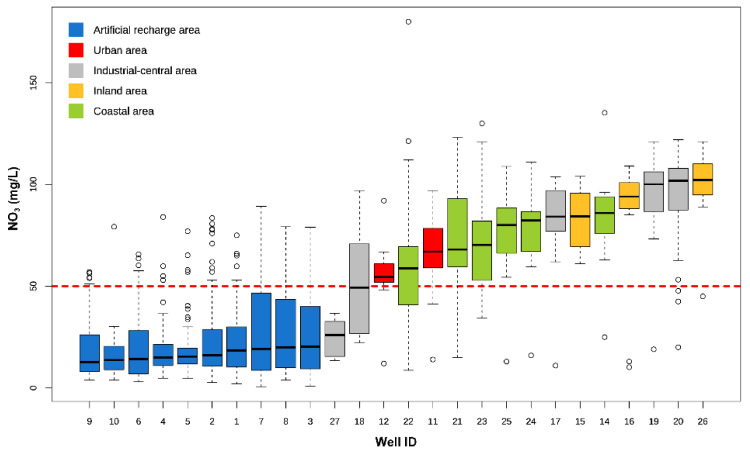
Boxplots of NO_3_^−^ concentration in mg/L measured in the monitored wells and ordered according to increasing median values. The horizontal dotted red line marks the Law Limit of 50 mg/L defined by the Italian legislation.

Marked differences are recognized in the NO_3_^−^ contents between the ARA and AP waters. In ARA, nitrate is mostly lower than 21 mg/L with a median value of 13.0 mg/L, while in AP the concentrations of NO_3_^−^ are significantly higher, being predominantly comprised between 58 and 94 mg/L with peaks up to 180 mg/L, and median of 76.2 mg/L. Boxplots of Figure 4 illustrate NO_3_^−^ variability in the monitored wells located in the different areas of the Metauro River plain. The NO_3_^−^ concentrations in ARA ranged from 5 to 84 mg/L (Table 2), with 93% of the water samples lower than the legislative threshold value of 50 mg/L. No significant differences are recognized when the whole dataset is considered (Table 1), where minimum and maximum values are 0.6 and 89.3 mg/L, respectively, with the 90% of the samples with NO_3_^−^ < 50 mg/L. Median values are always below 50 mg/L (Figure 4), ranging between 15.0 and 20.3 mg/L (see Supplementary Material), and exhibit a lower variability compared to that of other wells (Figure 3 and Figure 4), excepting for #3, #7 and #8 which are characterized by a higher variability over time. In the AP waters the great majority (85–88%) of the samples, both considering the balanced analyses (Table 2) or the whole dataset (Table 1), exceed the threshold value of 50 mg/L. The medians of the AP wells (between 54.2 and 102.0 mg/L), except for #18 (median = 49.3 mg/L) and #27 (median = 26.0 mg/L), overcome the law limit as they reach contents clustering around 100 mg/L in some wells located in the inland and industrial-central areas (#16, #19, #20, #26; Figure 4). Wells from urban and coastal areas show intermediate NO_3_^−^ median values (i.e., between 54.5 and 67.0 mg/L, and 58.7 and 86.0 mg/L, respectively, with the latter showing the highest variability (i.e., #21, #22, #23).

### 4.3. Temporal Trend Evolution of Nitrates

The trend analysis did not include #13, #14 and #27 due to the insufficient number of available observations (Table 3). According to the results of the MK test displayed in Table 3, nearly all NO_3_^−^ time series have decreasing concentration trends (*p*-value < 0.01). The sole exceptions are #12 and #19, for which there is no evidence of significant monotonic trends (*p*-value > 0.05), thus preventing them from being investigated via trend analysis. For those wells having meaningful NO_3_^−^ trends, Median-Based Linear Regression Models were applied, and the results of linear fitting (slope, intercept, *p*-values and residual standard error) are reported in Table 3. The applied methods provide reliable results as confirmed by the *p*-values below 0.01, and the most significant statistics are obtained for the Theil-Sen single median model. In general, the Siegel repeated median model produces slightly higher slope and intercept estimates than those resulting from the Theil-Sen model. The residual standard error (RSE) indicates that the Siegel model can predict NO_3_^−^ concentrations with about ±16.1 mg/L error on average compared to the ±15.6 mg/L of Theil-Sen model. Both models produce consistent results with small differences; nevertheless, the latter is chosen as the preferred model for its precautionary results and lower RSE. Wells located in the ARA (#1–10) have a median slope_Theil-Sen_ of −0.24, with the higher slopes (about −0.35) obtained for #7 and #8 (Table 3). The time evolution of the wells within urban areas diverges: #12 shows a stationary response, whereas #11 exhibits decreasing dynamics with a slope_Theil-Sen_ of −0.37. Similarly, the industrial-central area wells display heterogeneous responses: #19 shows a stationary pattern, #17 and #20 a decreasing trend (slope_Theil-Sen_ around −0.2) and #18 a relevant decrement (slope_Theil-Sen_ = −0.83). The inland area wells present slight downward trends with slopes around −0.2, except for #15 which has a higher slope (−0.36). Finally, the coastal wells record the highest reductions over the investigation area with a median slope_Theil-Sen_ of −0.52 and a maximum decrease observed for #23 (slope_Theil-Sen_ = −0.98). The BP test results, performed on the Theil-Sen model fitting, indicate that the model residuals are homoscedastic (*p*-value > 0.05) for most investigated wells (Table 3). By contrast, the null hypothesis that the variance of the residuals is constant is rejected for wells #6, #9, #18, #15, #22 and #23, indicating the presence of heteroscedasticity.

## 5. Discussion

### 5.1. Nitrate Sources Interpreted by Geochemical Indicators

The water chemistry of the shallow coastal aquifer of the terminal portion of the Metauro River plain has been comprehensively explored by Nisi et al. [18] through a detailed hydrogeochemical and isotopic study. Its origin is meteoric, and it is mainly fed by the Metauro River waters coupled with local infiltration waters and those transmitted from the western hills bordering the valley. The chemical compositions are dominantly calcium bicarbonate. They mainly originate through interaction with carbonate minerals, extensively present in the alluvial deposits of the plain. However, enrichments in Na^+^, and to a less extent in K^+^, are found in most of the investigated samples, especially in those from the coastal area (Figure 2).

**Figure 5:**
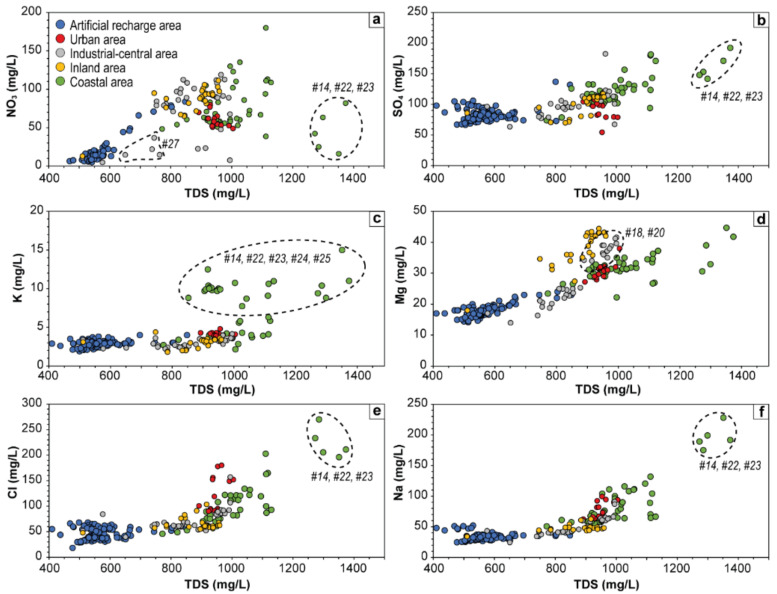
Binary diagrams of (**a**) NO_3_^−^, (**b**) SO_4_^2−^, (**c**) K^+^, (**d**) Na^+^, (**e**) Cl^−^ and (**f**) Mg^2+^ vs. TDS (in mg/L) in the shallow groundwater system of the Metauro River plain.

Besides water-rock interaction and secondary processes (e.g., ionic exchange, evaporation), the chemical composition of groundwater in agricultural and urbanized areas can largely be controlled by inputs of anthropic activities deriving from both pollution and land use [3,14,18,60,61,62,63,64,65]. Among the numerous chemical compounds, NO_3_^−^, SO_4_^2−^, K^+^, Cl^−^, and Na^+^ are frequently derived from agricultural fertilizers, industrial and municipal sewage, and animal waste [66,67] and their concentrations plotted against the TDS may indicate the influence of human activities on water chemistry [61]. These relationships, along with that with Mg^2+^, based on its influence on the groundwater chemistry of the inland area (Figure 3), are displayed in Figure 5. The ARA waters, with a few rare exceptions, generally show constant values of all six main ions in a relatively narrow TDS range (Table 2) and reflect a relatively homogeneous composition.

**Figure 6:**
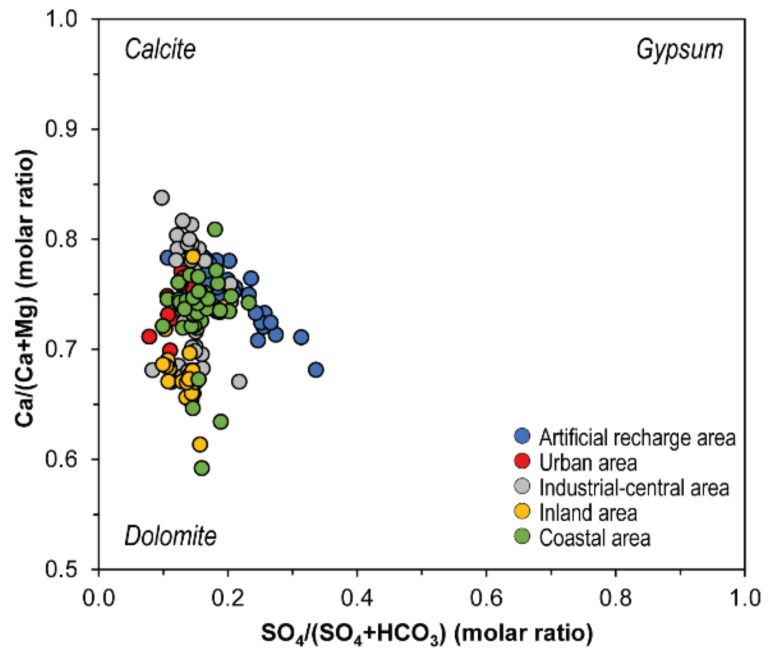
Ca/(Mg+Ca) vs. SO_4_/(SO_4_ − HCO_3_) diagram (after Frondini [72]).

Waters from the alluvial plain show strong enrichments of NO_3_^−^ at increasing salinity, with only samples from #27 located about 1.5 km downflow ARA (Figure 1d), that diverts from this trend showing nearly constant value of NO_3_^−^ (Figure 5a). Virtually all the samples exceed 3 mg/L, i.e., the threshold value above which the anthropogenic influence is occurring [68,69,70]. Sulfate shows slight increments for increasing TDS (Figure 5b). Potassium shows values ranging in a narrow range (2–6 mg/L), excluding wells located in the coastal area (Figure 5c). The concentration of K^+^ in the latter wells (except for #21) is mainly comprised between 9 and 15 mg/L and is likely responsible for the increased variability of the K^+^ log-ratio resulting from the biplot of Figure 3b. The molar ratio with the other main ions does not show any specific correlation, suggesting an anthropogenic input linked to an excessive use of K-fertilizers [71]. Magnesium shows a general constant positive trend with increasing TDS contents (Figure 5d). In this case, all samples from the inland area and some of those from the industrial-central area (#18 and #20) divert from this trend, showing enrichments in Mg^2+^ (up to 43 mg/L) with respect to samples in the same TDS range (i.e., ~800–1000 mg/L). Figure 6 shows the Ca/(Ca+Mg) vs. SO_4_/(SO_4_+ HCO_3_) molar ratios, with ordinate axis ranging from 1, corresponding to the dissolution of pure calcite, to 0.5, corresponding to the dissolution of stoichiometric dolomite and abscissa ranging from 0 to 1, corresponding to dissolution of stoichiometric dolomite/calcite and gypsum, respectively [72]. Samples from AP are characterized by values of Ca/(Ca+Mg) molar ratios generally comprised between 0.85 and 0.7 indicating that groundwater reacts with both calcite and dolomite. Samples with higher Mg^2+^, however, show ratios < 0.7, suggesting that dedolomitization driven by dissolution of gypsum or anhydrite layers [72,73] presents in the local bedrock (e.g., Gessoso-Solfifera Fm; Figure 1c) occur.

**Figure 7:**
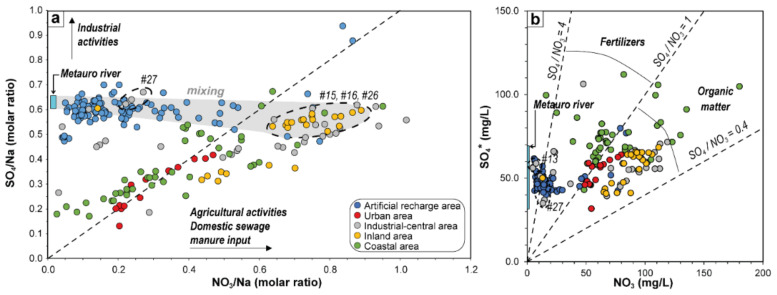
(**a**) Binary diagram of SO_4_/Na vs. NO_3_/Na (as molar ratios) for the investigated samples, where the dashed line represents a 1:1 ratio. (**b**) Plot of SO_4_* vs. NO_3_, where the dashed lines define the different ratios discriminating the sources of pollution (after Busico et al. [63], Cuoco et al. [76] and Rufino et al. [77]). Metauro River fields from Nisi et al. [18].

Both Cl^−^ and Na^+^ display slight, or almost null, increments in most of the samples up to TDS ~950 mg/L (Figure 5e,f). However, at higher TDS values more marked increments are displayed. The coastal waters generally show different characteristics with respect to the other wells located in AP. In fact, they generally show higher nitrate contents at lower TDS values, roughly resembling a negative trend (Figure 5a). Besides HCO_3_^−^ and Ca^2+^, the TDS of these wells is mostly controlled by Cl^−^ and Na^+^, and to a lesser extent by SO_4_^2−^ (Figure 7b). This is particularly evident in those wells showing TDS > 1250 mg/L, i.e., #14, #23, #24. Considering the proximity of these latter wells to the coastline (Figure 2), the enrichments in Na^+^ and Cl^−^ might be related to seawater intrusion, which could lead to an increase in both ions. However, Nisi et al. [18] found that the shallow aquifer of the Metauro River plain is not apparently affected by salinization, thus suggesting that the higher values in Na^+^ could be more likely related to dissolution processes suffered by silicate minerals present in the local substrate (Figure 1c). The higher Cl^−^ concentrations and the significant variability of this ion in the Cl^−^ log-ratio for both ARA and AP groundwater (Figure 3b,c), on the contrary, seem to be consistent with an anthropogenic source (see below).

Bivariate plots of selected ion molar ratios are often used for exploring possible nitrate pollution sources and tracing the influence of natural and anthropogenic processes [3,14,65,66,67,74]. The SO_4_/Na and NO_3_/Na molar ratios can be used to preliminarily evaluate the presence of anthropogenic inputs [65]. The studied waters depict two clear and distinct trends when these ratios are plotted against each other (Figure 7a). AP samples show a generally positive correlation between both ratios, with NO_3_/Na and SO_4_/Na comprised between ~0.2 and 1 and ~0.1 and 0.65, respectively. Most samples display a shift from the 1:1 line, suggesting that NO_3_^−^ predominates on SO_4_^2−^ due to a major contribution of domestic sewage and/or manure and agricultural inputs with respect to industrial activities (Figure 7a). The ARA waters display quite constant SO_4_/Na (~0.6) ratios, whereas that of NO_3_/Na is much more variable: from 0.04 (i.e., like that of the Metauro River waters: 0.02–0.03; [18]) up to 0.84. The SO_4_/Na ratios in these waters approach the Metauro River water ratio (0.62–0.67; [18]), reflecting mixing processes between ground- and surface- waters used to recharge and dilute the shallow aquifer. The dilution effect on the nitrate contents, which supports the effectiveness of the artificial recharge system, seems to be confirmed by the fact that similar SO_4_/Na ratios and remarkably higher NO_3_/Na ratios are recorded in those wells (#15, #16 and #26; Figure 7a) located upstream the ARA wells (Figure 1d), while lower NO_3_/Na ratios (at similar SO_4_/Na) are measured downstream the groundwater flow from ARA (#27; Figure 1d and Figure 7a). The SO_4_/Na ratio in the ARA and Metauro waters is generally higher than those observed in the water samples from the shallow coastal aquifer, suggesting either the contribution from natural gypsum-rich formations (e.g., evaporites), which are present along the inner parts of the river course ([21]; not shown in Figure 1c) or the presence of effluents from industrial activities [65]. However, the former seems to be of relevance when the ARA waters are considered, given the higher values (up to 0.4) of the SO_4_/(SO_4_+ HCO_3_) molar ratio (Figure 6).

To further explore the sources of SO_4_^2−^ and NO_3_^−^, both anions (in mg/L) are plotted against each other in Figure 7b. In this case, to eliminate the evaporitic contribution likely related to SO_4_^2−^ as CaSO_4_, at the total concentration of Ca^2+^ (in meq/L) the stoichiometrically equal quantity of sulfate was subtracted. The computed result was then multiplied by the equivalent weight of SO_4_^2−^ to obtain SO_4_^2−^*, expressed in mg/L. Except for the coastal area samples that show a scattered behavior, a roughly positive correlation between the two dissolved species is present in the AP samples. Most waters have SO_4_/NO_3_ ratio comprised between 0.4 and 1, thus suggesting that the main anthropic contribution of the two pollutants derives from organic pollution, possibly linked to urban and rural sewage, animal waste and septic tanks [63,75,76,77]. Some samples, mostly located in the coastal area, have an SO_4_/NO_3_ ratio between 1 and 4 indicating that agricultural chemical fertilizers are likely impacting the groundwater [63,76,77] in this part of the Metauro River plain. In most ARA and #13 and some #27 (AP) samples, sulfate dominates over nitrate (mean ratio 4.1 and maximum values up to 11.3), approaching that recorded for the Metauro River waters (SO_4_/NO_3_ > 16; [18]).

**Figure 8:**
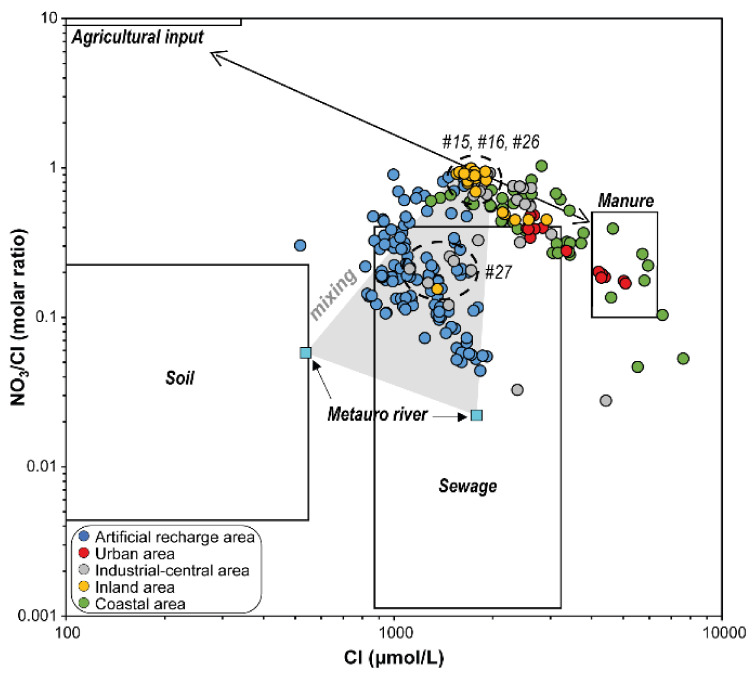
Variation in Cl^−^ concentrations (in μmol/L) with respect to NO_3_/Cl molar ratios of groundwater samples collected from the shallow coastal aquifer of the Metauro River plain. Representative fields of agricultural, soil, sewage and manure inputs are from Torres-Martínez et al. [6]; Metauro river samples are from Nisi et al. [18].

Co-variation in the NO_3_/Cl molar ratios with concentration of Cl^−^ gives further insights on the origin of nitrate [6,69,78,79,80,81]. Generally, chemical fertilizers have high NO_3_^−^ with low Cl^−^ contents, while sewage waste and manure display high Cl^−^ concentrations and low NO_3_/Cl ratios [6,78,79,80,82,83,84]. The use of this plot requires that the effect of halite must be considered [84], although it seems to have a minor effect on the geochemical composition of the Metauro River plain groundwater [18]. In Figure 8, two trends can be observed for the AP and ARA samples, respectively. The NO_3_/Cl ratios in the AP waters are largely variable (from 0.03 to 1.03), with Cl^−^ values ranging from ~1000 up to ~7500 μmol/L (Figure 8). These samples are aligned along a theoretical trend between agricultural and manure inputs, with a prevalence of the latter, given that virtually all the samples show NO_3_/Cl ratios < 1 [80]. The ARA waters show NO_3_/Cl ratios comparable to those of the AP, but with lower Cl^−^ contents, with most of the samples falling inside the sewage field (Figure 8). Looking at the diagram, a possible mixing between agricultural fertilizers and sewage seems to be conceivable, as suggested by those samples with NO_3_/Cl ratios > 0.6 that slightly tend toward the agricultural fertilizers field. Nevertheless, these waters mostly fall inside a theoretical mixing field formed by the inland samples (#15, #16, #26) with the Metauro River waters used to dilute the aquifer. In this case, the Cl^−^ concentrations are between 1000 and 2000 μmol/L, while the NO_3_/Cl molar ratios range from the highest values (i.e., ~1) down to 0.04, the latter similar to that of the river waters (i.e., 0.02–0.06; [18]). Even in this case, samples from #27 fall between the inland and ARA samples (Figure 8), showing values of Cl^−^ and NO_3_/Cl molar ratios similar to the latter (i.e., Cl^−^ ~1100–1800 μmol/L; NO_3_/Cl ~0.2–0.3).

### 5.2. Nitrates Temporal Trend Analysis

**Figure 9:**
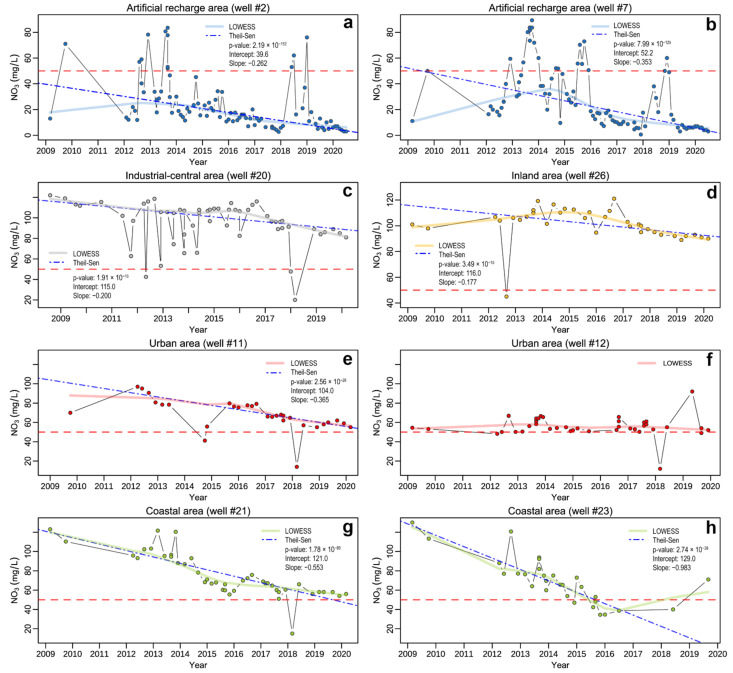
Nitrates time series from eight selected ((**a**) well #2; (**b**) well #7; (**c**) well #20; (**d**) well #26; (**e**) well #11; (**f**) well #12; (**g**) well #21; (**h**) well #23) wells located in the different areas of the alluvial plain, with the following fitting lines superimposed: LOWESS smoother (line), Theil-Sen fit (blue dash-dotted line). Summary statistics of the Theil-Sen median-based linear regression models are reported in each plot. The horizontal red dotted line marks the Law Limit of 50 mg/L defined by the Italian Legislation.

**Figure 10:**
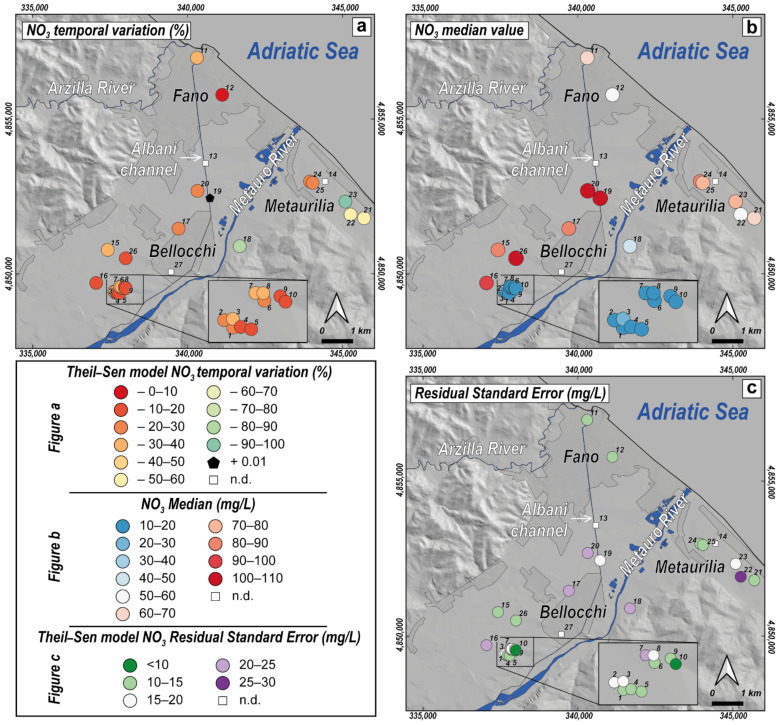
(**a**) Temporal variations in NO_3_^−^ concentrations (in %) defined by the Theil-Sen model; (**b**) NO_3_^−^ median values (in mg/L); (**c**) Residual Standard Error (in mg/L) defined by the Theil-Sen model. All the maps are referred to data belonging to the 2009–2020 span-time.

In order to recharge and dilute the phreatic aquifer and reduce the nitrate concentration, the local water supply company installed an artificial groundwater reinjection plant in the early 1990s, where the Metauro River waters are directly pumped into the aquifer. Although in many wells the nitrate contents still remain high (i.e., >50 mg/L), a general decrease can be detected by the temporal trends analysis (Table 3) with nearly all NO_3_^−^ time series showing significantly decreasing concentration trends. This behaviour is also visible in the time series plots (from 2009 up to 2020) of Figure 9, where selected wells, spatially dislocated in the alluvial plain, are reported along with the LOWESS smoother and Theil-Sen fitting line. In the maps of Figure 10, the spatial visualization of NO_3_^−^ temporal variation, median and RSE (referred to the Theil-Sen model) is illustrated. The time plots of ARA wells (Figure 9a,b) show that most observations are well predicted by the median model, indicating a reduction in NO_3_^−^ concentrations by 24–35% over the analyzed time period (Figure 10a). As highlighted by the LOWESS smoother, this decrease seems more pronounced since 2016. However, many values, mainly occurring during 2012–2013 and in 2018, positively deviate from the linear regression model. These sporadic peaks can likely be related to either different injection rates of fluvial waters in the aquifer or temporary increases in NO_3_^−^ concentrations in the Metauro River, possibly deriving from runoff of agricultural or urban areas, being surface waters more sensitive to chemical variations with respect to groundwater (e.g., [85]). These wells also have a relatively high RSE compared to the NO_3_^−^ median, thereby confirming a high variability around the trend (Figure 10b,c). This fact, coupled with the evidence of heteroscedasticity of residuals, emerged from the BP test in some ARA wells (Table 3) and suggests that the model cannot fully explain the variability of the parameters. Therefore, the results should only be used for descriptive purposes or general frames of reference, and not for predicting future concentrations. To gain a better understanding of the processes behind the observed variability, a more detailed study with continuous monitoring is necessary to obtain suitable data for reliable predictions. The same is also true for the other monitored wells for which heteroscedasticity of residuals is detected.

Despite the limited available data (Table 3), waters from well #27 show NO_3_^−^ concentrations always below 50 mg/L (Figure 4), thus suggesting that the artificial recharge process is particularly effective up to the southwestern part of the Bellocchi industrial area (~1.5 km downflow the ARA; Figure 1d). Nevertheless, despite maintaining significantly high median values, the time-plot of #20 (Figure 9c) reveals that the deviations from the Theil-Sen model are mostly negative. This, coupled with the downward trends of approximately 20% with acceptable RSEs observed in #17 and #20 (Figure 10), indicates that the dilution effect related to the reinjection plant might still be present at larger distances, although much less marked. Similarly, wells located in the inland area also exhibit a decrease between 18 and 36% (Figure 10a), but with relatively lower RSE values compared to the highest medians, ranging between 90 and 100 mg/L (Figure 4 and Figure 10b). The model is thus providing better predictions, as also evidenced by the time-plot in Figure 9d. Typically, both inland and industrial-central wells experienced a greater reduction in NO_3_^−^ from 2015, as shown by the LOWESS smoother lines (Figure 9c,d). The time evolution of #11 (Figure 9e) in the urban area appears to be in good agreement with wells monitored in the inland and industrial-central areas, with a few minor negative deviations from the model. However, this behaviour is not consistent with that observed in #12 (Figure 9f), which has a lower median value and an almost stationary pattern. It is not easy to explain these differences. Both wells are indeed located within the urban agglomeration, on an alluvial deposit that has similar thickness (Figure 1c) and comparable saturated thickness [18,22]. However, #11 is located in the northern part of Fano, at the border of the Arzilla River basin, classified as a nitrate vulnerable zone (Figure 1b). We can only speculate that the water chemistry of this aquifer can influence that of #11 since, to the best of our knowledge, no data about the aquifer zone close to the Arzilla River are available. As depicted in Figure 10a, the highest decreases in NO_3_^−^ concentrations (50–98%) occurred along the hydrographic right of the Metauro River (#18) and in the coastal area of Metaurilia. Noteworthy, the downward trend in the latter is characterized by an important decreasing rate until 2015 (Figure 9g,h), much earlier than in the other monitored wells where the NO_3_^−^ decrease becomes more evident starting from 2016–2017. Interestingly, these areas are not directly influenced by any artificial recharge. This may imply that different driving processes are affecting the nitrate response in the two areas separated by the Metauro River.

## 6. Conclusions

The NO_3_^−^ pollution is a long-lasting story in the Metauro River valley (as well as in many other alluvial plains of Italy), since nitrate started to increase in the 1970s. Currently, an artificial recharge system of the aquifer, implemented by the local water management company, injects low-NO_3_^−^ Metauro River waters directly into the aquifer, thereby causing a dilution of nitrate concentrations.

In this study, geochemical and robust compositional principal component analyses were combined to analyze the water composition of the shallow coastal aquifer of the Metauro River plain. Our results show that near the artificial recharge area, the groundwater composition appears to be quite homogeneous, while in the alluvial plain additional sources of analytes are evident. Nitrate and Cl^−^ have the largest influence on the groundwater composition variability. The major local sources of both analytes, along with SO_4_^2−^, in the study area, seem to be linked with organic pollution likely related to manure input used in local agricultural practices. Additional inputs from urban/rural sewage, animal waste and septic tanks seems to be conceivable, whilst agricultural chemical fertilizers appear to be less impactful. However, inputs related to an excessive use of K-fertilizers affecting the coastal area have been recognized.

The time trends of NO_3_^−^ data collected over a 12-year period reveal that the artificial recharge system is highly effective up to ~1.5 km downflow of the injection system producing a decrease in NO_3_^−^ concentrations over time. Nevertheless, NO_3_^−^ shows a general decrease throughout the years, even in those areas not directly affected by the artificial recharge, with the greatest reduction observed in the coastal area of Metaurilia. The generalized decrements in the NO_3_^−^ contents in different sectors of the alluvial plain located far from the injection area are possibly due to the national, regional, and local regulations, which limit the periods and quantities of fertilizers that can be used on the territory since the early 2000s (e.g., the WFD). These actions are positively improving the water quality; however, almost all the sampled wells still show median concentrations of NO_3_^−^ above the law limit (50 mg/L), suggesting that the effects will likely be observed at medium-to-long term.

It is a matter of fact that an isotopic signature of nitrate (δ^15^N and δ^18^O) and a quantitative estimate of the contribution of natural and anthropogenic nitrate sources is required, to better define sources and processes affecting the N-compounds in groundwater and intervene with specific actions to further reduce the nitrate pollution.

## Figures and Tables

**Table 1:** Main summary statistics of the available data for the ARA (upper panel) and AP (lower panel) monitored wells, respectively. N. Obs. = number of available measurements; Min = minimum value; Max = maximum value; Mean = average value; Median = median value; SD = standard deviation. Data are expressed in mg/L.

**Artificial Recharge Area (ARA) Wells**						
**Parameter**	**N. obs.**	**Min**	**Max**	**Mean**	**Median**	**SD**
HCO_3_	118	123	388	272	273	39.9
Cl	158	18.4	68.0	43.1	40.3	10.5
NO_3_	926	0.60	89.3	22.0	16.1	17.7
NO_2_	12	0.01	0.01	0.01	0.01	0.00
SO_4_	158	65.6	137	82.9	81.0	11.0
Na	158	24.8	52.0	33.7	32.9	5.08
NH_4_	2	0.40	19.0	9.70	9.70	13.2
K	148	1.90	4.00	2.82	2.80	0.37
Mg	158	14.2	24.0	18.0	17.8	1.84
Ca	158	60.0	143	94.0	91.4	11.9
SiO_2_	18	5.00	15.0	10.4	11.0	2.55
pH	158	7.00	8.21	7.54	7.53	0.27
TDS	118	410	848	564	555	63.2
EC	158	517	907	657	649	62.1
**Alluvial Plain (AP) Wells**						
**Parameter**	**N. obs.**	**Min**	**Max**	**Mean**	**Median**	**SD**
HCO_3_	121	162	573	410	416	55.8
Cl	222	39.5	366	98.0	84.4	49.8
NO_3_	544	4.82	180	76.7	80.4	25.8
NO_2_	26	0.01	0.42	0.05	0.01	0.08
SO_4_	222	52.0	197	113	109	30.2
Na	205	24.5	313	70.7	61.0	40.8
NH_4_	64	0.01	2.00	0.28	0.01	0.54
K	202	1.70	15.0	4.76	3.50	2.97
Mg	204	12.5	44.7	31.5	31.9	7.27
Ca	190	58.9	178	138	142	20.7
SiO_2_	31	9.00	19.0	14.2	15.0	2.99
pH	205	6.70	8.14	7.24	7.19	0.22
TDS	120	511	1374	926	936	135
EC	211	603	2020	1095	1067	197

**Table 2:** Descriptive statistics related to the subset of complete and balanced analyses for ARA (upper panel) and AP (lower panel) monitored wells, respectively. N. Obs. = number of available measurements; Min = minimum value; Max = maximum value; Mean = average value; Median = median value; SD = standard deviation; MAD = median absolute deviation; Skew = third standardized moment; Kurtosis = fourth standardize moment; 25% (Q1) = 1st quartile value; 75% (Q3) = 3rd quartile value. Data are expressed in mg/L and observations <DL were previously replaced with the value of the detection limit.

**Artificial Recharge Area (ARA) Wells**											
**Parameter**	**N. obs.**	**Min**	**Max**	**Mean**	**Median**	**SD**	**MAD**	**Skew**	**Kurtosis**	**25% (Q1)**	**75% (Q3)**
HCO_3_	118	123	388	272	273	39.9	16.5	−0.94	2.83	265	290
Cl	118	18.4	68.0	44.7	41.8	10.7	10.6	0.34	−0.82	36.5	53.8
NO_3_	116	5.00	84.0	18.8	13.0	16.4	8.08	2.14	4.45	8.68	21.0
SO_4_	118	66.7	137	82.9	80.7	10.9	7.93	1.99	6.59	76.0	87.0
Na	118	24.8	52.0	33.6	32.5	5.53	3.78	1.50	2.42	30.0	35.3
K	118	1.90	4.00	2.86	2.90	0.39	0.30	0.28	0.48	2.60	3.08
Mg	118	14.2	24.0	17.7	17.3	1.85	1.11	1.01	1.56	16.7	18.4
Ca	118	60.0	143	92.7	90.0	12.4	7.41	1.35	3.4	86.3	96.5
pH	118	7.00	8.21	7.62	7.60	0.26	0.24	−0.15	−0.06	7.47	7.82
TDS	118	410	848	565	555	63.5	36.8	1.70	5.26	533	580
EC	118	517	907	654	647	64.5	40.0	1.37	3.28	620	670
**Alluvial Plain (AP) Wells**											
**Parameter**	**N. obs.**	**Min**	**Max**	**Mean**	**Median**	**SD**	**MAD**	**Skew**	**Kurtosis**	**25% (Q1)**	**75% (Q3)**
HCO_3_	117	217	573	411	416	49.7	28.4	−0.76	3.96	386	432
Cl	117	39.5	270	91.9	82.0	43.0	31.1	1.70	2.92	62.0	107
NO_3_	117	4.82	180	74.5	76.2	29.6	27.0	−0.03	0.68	58.0	94.0
SO_4_	117	54.5	192	108	107	25.8	17.6	0.84	1.31	95.0	118
Na	117	24.5	228	69.0	60.3	34.8	21.3	2.26	6.07	46.8	76.2
K	117	1.80	15.0	4.79	3.60	2.90	1.04	1.41	0.79	3.05	4.39
Mg	117	14.0	44.7	31.8	31.8	7.05	6.52	−0.30	−0.34	27.9	36.3
Ca	117	84.0	178	141	144	20.0	21.5	−0.66	0.15	127	155
pH	117	7.00	8.03	7.33	7.30	0.21	0.18	1.04	1.38	7.18	7.42
TDS	117	511	1374	932	937	136	85.6	0.21	2.54	859	990
EC	117	603	1704	1086	1062	181	132	0.72	2.08	979	1158

**Table 3:** Results of the linear fitting analysis. The following parameters are reported: well ID; total number of available measurements (N. Obs.); median-based Siegel test parameters (Slope_Sieg_, Intercept_Sieg_, *p*-value_Sieg_, Residual Standard Error = RSE_Sieg_); Theil-Sen single median test parameters (Slope_Theil-Sen_, Intercept_Theil-Sen_, *p*-value_Theil-Sen_, RSE_Theil-Sen_); *p*-values of the Mann-Kendall test (*p*-value_MK_) and Breusch–Pagan test (*p*-value_BP_).

ID	N. Obs.	Slope_Sieg_	Intercept_Sieg_	*p*-Value_Sieg_	RSE_Sieg_	Slope_Theil−Sen_	Intercept_Theil−Sen_	*p*-Value_Theil−Sen_	RSE_Theil−Sen_	*p*-Value_MK_	*p*-Value_BP_
ARA wells											
1	92	−0.29	43.7	9.3 × 10^−12^	14.7	−0.27	42.3	1.7 × 10^−121^	14.5	2.2 × 10^−10^	0.541
2	93	−027	38.2	7.4 × 10^−13^	19.2	−0.26	39.6	2.2 × 10^−152^	18.6	6.9 × 10^−12^	0.103
3	94	−0.34	47.3	8.9 × 10^−13^	19.1	−0.32	47.9	3.5 × 10^−129^	18.3	1.3 × 10^−10^	0.479
4	93	−0.13	24.9	1.1 × 10^−9^	13.4	−0.12	24.3	5.7 × 10^−65^	13.3	5.5 × 10^−6^	0.151
5	93	−0.10	24.0	7.6 × 10^−10^	13.2	−0.12	24.9	4.7 × 10^−83^	13.2	6.3 × 10^−7^	0.580
6	92	−0.24	34.1	6.9 × 10^−15^	14.6	−0.22	34	5.2 × 10^−133^	14.1	1.9 × 10^−11^	1.5 × 10^−4^
7	93	−0.41	55.3	3.0 × 10^−15^	22.6	−0.35	52.2	8.0 × 10^−125^	21.8	3.4 × 10^−11^	3.2 × 10^−4^
8	93	−0.44	56.9	3.1 × 10^−15^	20.9	−0.35	52.9	5.7 × 10^−118^	19.4	1.7 × 10^−10^	2.5 × 10^−6^
9	92	−0.19	28.9	2.5 × 10^−14^	13.6	−0.15	26.2	5.0 × 10^−98^	13.5	3.7 × 10^−9^	2.0 × 10^−5^
10	93	−0.16	27.5	5.3 × 10^−14^	9.19	−0.13	24.3	2.9 × 10^−122^	9.21	5.9 × 10^−9^	0.298
AP wells—urban area											
11	30	−0.39	109	2.1 × 10^−7^	15.5	−0.37	104	2.6 × 10^−26^	14.5	8.1 × 10^−6^	0.699
12	41	-	-	-	-	-	-	-	-	6.5 × 10^−1^	0.107
AP wells—industrial-central area											
13	5	-	-	-	-	-	-	-	-	-	-
17	17	−0.39	129	4.6 × 10^−5^	30.6	−0.23	106	3.8 × 10^−7^	21.9	9.5 × 10^−3^	0.483
18	26	−0.97	99.6	8.8 × 10^−6^	22.3	−0.83	93.6	2.2 × 10^−20^	20.4	5.9 × 10^−4^	2.4 × 10^−2^
19	38	-	-	-	-	-	-	-	-	5.3 × 10^−1^	0.284
20	55	−0.18	116	6.6 × 10^−4^	21.5	−0.20	115	1.9 × 10^−16^	21.0	1.2 × 10^−3^	0.888
27	10	-	-	-	-	-	-	-	-	-	-
AP wells—inland area											
15	36	−0.41	115	4.1 × 10^−7^	12.2	−0.36	111	2.0 × 10^−16^	11.4	5.7 × 10^−6^	1.9 × 10^−2^
16	34	−0.18	110	7.5 × 10^−6^	21.5	−0.19	111	2.0 × 10^−22^	21.5	1.1 × 10^−5^	0.763
26	35	−0.25	126	2.1 × 10^−5^	14.7	−0.18	116	3.5 × 10^−15^	13.3	1.3 × 10^−3^	0.244
AP wells—coastal area											
14	9	-	-	-	-	-	-	-	-	-	-
21	44	−0.62	120	7.9 × 10^−9^	13.4	−0.55	121	1.8 × 10^−89^	12.5	4.4 × 10^−12^	0.712
22	46	−0.36	85.8	2.4 × 10^−5^	27.9	−0.52	106	2.3 × 10^−27^	27.3	7.7 × 10^−4^	4.5 × 10^−4^
23	30	−1.02	132	3.7 × 10^−9^	18.1	−0.98	129	2.7 × 10^−39^	18.0	1.6 × 10^−7^	3.1 × 10^−2^
24	44	−0.36	106	7.2 × 10^−8^	14.0	−0.27	101	2.5 × 10^−27^	14.5	5.8 × 10^−4^	0.219
25	43	−0.33	106	5.4 × 10^−10^	14.5	−0.27	101	5.4 × 10^−27^	14.4	5.9 × 10^−4^	0.086

## Data Availability

Data are available upon request to the corresponding author.

## Supplementary Materials

The following supporting information can be downloaded at: https://www.mdpi.com/article/10.3390/ijerph191912231/s1, Table S1: main statistical parameters of compositional data for each well; Figure S1: boxplots of the main physicochemical parameters; Table S2: nitrate contents statistical parameters for each investigated well.
