# Supplementary material for: Contamination Assessment and Temporal Evolution of Nitrates in the Shallow Aquifer of the Metauro River Plain (Adriatic Sea, Italy) after Remediation Actions
Source: Int J Environ Res Public Health. 2022 Sep 27;19(19):12231. doi: 10.3390/ijerph191912231 (PMC9566390; doi:10.3390/ijerph191912231)
Supplement: Supplementary file 1 [file ijerph-19-12231-s001.zip › ijerph-1876158-supplementary.pdf]

Contamination assessment and temporal evolution of nitrates in the shallow aquifer of the Metauro River plain (Adriatic Sea, Italy) after remediation actions

Marco Taussi [1,*], Caterina Gozzi [2], Orlando Vaselli [2,3], Jacopo Cabassi [3], Matia Menichini [4], Marco Doveri [4], Marco Romei [5], Alfredo Ferretti [5], Alma Gambioli [5], Barbara Nisi [3]

1 Dipartimento di Scienze Pure e Applicate, Università degli Studi di Urbino Carlo Bo, Via Ca' Le Suore 2/4, Urbino, Italy
2 Dipartimento di Scienze della Terra, Università di Firenze, Via G. La Pira 4, Firenze, Italy
3 CNR-IGG Istituto di Geoscienze e Georisorse, Consiglio Nazionale delle Ricerche, Via G. La Pira 4, Firenze, Italy
4 CNR-IGG Istituto di Geoscienze e Georisorse, Consiglio Nazionale delle Ricerche, Via G. Moruzzi 1, Pisa, Italy
5 ASET SpA, Via Luigi Einaudi 1, 61032 Fano, Italy;
* Correspondence: marco.taussi@uniurb.it

Below are reported the tables and the boxplots with the main statistical parameters of the physico-chemical composition and the nitrate contents for each investigated well.

Table S1: main statistical parameters of compositional data for each well.

Well #1 (ARA)						
Parameter	*N. Obs*	*Min*	*Max*	*Mean*	*Median*	*SD*
HCO_3	11	189	312	272	283	42.7
Cl	11	32.8	68	46.2	48	11.7
NO_3	11	6.5	66	22.8	18	18.7
SO_4	11	71.7	103	81.6	74.7	10.3
Na	11	28	50	34.2	34	6.17
NH_4	2	0.4	19	9.7	9.7	13.2
K	11	2.8	4	3.18	3.1	0.37
Mg	11	16.6	23	18.65	18.1	2.12
Ca	11	84.2	126	94.54	91	12.7
SiO_2	1	13	13	-	-	-
pH	11	7.26	8.12	7.62	7.58	0.28
TDS	11	509	695	575	570	58.4
EC	11	597	834	670	649	71.4

Well #2 (ARA)						
Parameter	*N. Obs*	*Min*	*Max*	*Mean*	*Median*	*SD*
HCO_3	12	195	345	277	278	37.7
Cl	12	30.6	65	44.9	43.3	12.7
NO_3	12	5	71	19.9	16.8	17.7
SO_4	12	68.7	106	83.5	85.5	11.9
Na	12	26.8	52	33.7	33.2	6.7
K	12	2.4	3.7	2.89	2.85	0.36
Mg	12	16.9	23	18.5	17.9	1.9
Ca	12	81.8	128	93.2	88.7	12.4
SiO_2	2	8	11	9.5	9.5	2.12
pH	12	7	7.9	7.55	7.56	0.24
TDS	12	516	744	574	564	61.2
EC	12	582	844	661.75	655	68.6

Well #3 (ARA)

Parameter	N. Obs	Min	Max	Mean	Median	SD
HCO_3	12	146	344	275	280	50.1
Cl	12	33.1	66	47	44.4	11.5
NO_3	12	6.4	79	24.8	16.4	21.9
SO_4	12	73.6	137	88.4	83.6	18.2
Na	12	28	51	34	33.4	6
K	12	2.5	3.5	3.02	3	0.32
Mg	12	16.6	22.4	18.6	18	1.88
Ca	12	73	129	94.8	91.4	15.32
SiO_2	2	9	13	11	11	2.83
pH	12	7	8	7.56	7.57	0.29
TDS	12	469	801	585	555	86.51
EC	12	592	852	672	649	74.7
Well #4 (ARA)						
Parameter	*N. Obs*	*Min*	*Max*	*Mean*	*Median*	*SD*
HCO_3	12	176	376	276	276	44.35
Cl	12	33.2	60.8	45.6	43.2	9.97
NO_3	12	7.5	84	18.5	12.3	21.1
SO_4	12	75.3	132	86.7	83.3	15.2
Na	12	26.7	46	33.8	33.6	4.76
K	12	2.31	4	2.95	3	0.45
Mg	12	15.3	23	17.6	17.2	2.05
Ca	12	78	135	93	88.7	14.3
SiO_2	2	9	14	11.5	11.5	3.54
pH	12	7	8.01	7.59	7.57	0.28
TDS	12	480	848	574	554	90.4
EC	12	586	885	658	639	77.3
Well #5 (ARA)						
Parameter	*N. Obs*	*Min*	*Max*	*Mean*	*Median*	*SD*
HCO_3	12	198	388	281	275	41.8
Cl	12	35.5	62	46.5	46.4	8.51
NO_3	11	8.5	77	20.9	14.2	19.4
SO_4	12	71.2	100	81.3	80	7.53
Na	12	29.4	47	34.5	33.9	4.55
K	12	2.16	3.2	2.86	2.9	0.25
Mg	12	14.6	24	17.7	16.9	2.36
Ca	12	85.8	143	95.1	89.5	15.94
SiO_2	2	10	15	12.5	12.5	3.54
pH	12	7.26	8	7.64	7.62	0.23
TDS	12	515	803	578	557	74.2
EC	12	617	907	668	641	80.2
Well #6 (ARA)						
Parameter	*N. Obs*	*Min*	*Max*	*Mean*	*Median*	*SD*
HCO_3	11	169	298	261	266	35.1
Cl	11	18.4	65	40.3	38.2	12.8
NO_3	11	5	28	12.2	9.7	8.05
SO_4	11	66.7	97	80.3	76.9	10.7
Na	11	24.8	44	32.5	30.5	6.14
K	11	2.11	3.7	2.68	2.6	0.5
Mg	11	15	19.1	16.8	17	1.38
Ca	11	68	97.8	85.9	85.7	9.17
SiO_2	1	11	11	-	-	-
pH	11	7.4	8.11	7.69	7.59	0.23
TDS	11	437	593	532	529	46.9

Parameter	N. Obs	Min	Max	Mean	Median	SD
EC	11	517	710	617	627	60
Well #7 (ARA)						
Parameter	*N. Obs*	*Min*	*Max*	*Mean*	*Median*	*SD*
HCO_3	12	175	329	277	278	41.7
Cl	12	29	68	42.7	40.5	12
NO_3	11	6	55.7	23.2	15.2	19.3
SO_4	12	67.8	104	82.8	79.8	9.94
Na	12	25.6	51	32.8	30.8	7.01
K	12	1.9	3.8	2.7	2.6	0.52
Mg	12	14.4	20.7	17.9	18.1	1.95
Ca	12	78	120	95.6	94.9	12.5
SiO_2	2	7	11	9	9	2.83
pH	12	7	8.11	7.6	7.59	0.31
TDS	12	493	668	572	566	61.5
EC	12	556	752	660	663	63.6
Well #8 (ARA)						
Parameter	*N. Obs*	*Min*	*Max*	*Mean*	*Median*	*SD*
HCO_3	12	123	320	270	278	50.4
Cl	12	32.5	57	44.2	43.1	8.65
NO_3	12	5	45.4	20.3	13.5	14.91
NO_2	8	0.01	0.01	0.01	0.01	0
SO_4	12	73.4	98	82	81.2	6.43
Na	12	28.1	48	33.2	32	5.2
K	12	2.09	3.2	2.69	2.75	0.35
Mg	12	14.2	19.5	17.1	17	1.52
Ca	12	60	109	92.2	93.1	13.2
SiO_2	2	5	11	8	8	4.24
pH	12	7	8	7.59	7.61	0.29
TDS	12	410	648	561	561	64.6
EC	12	521	731	651	658	62
Well #9 (ARA)						
Parameter	*N. Obs*	*Min*	*Max*	*Mean*	*Median*	*SD*
HCO_3	12	206	320	268.56	267.6	28.06
Cl	12	29.4	62	44.65	42.35	11.01
NO_3	12	5	31.7	12.8	10.7	7.83
SO_4	12	70.7	98	81.99	81.35	7.15
Na	12	27.3	48	33.45	32.25	5.38
K	12	2.1	3.1	2.73	2.75	0.33
Mg	12	14.47	18.5	16.99	17.25	1.27
Ca	12	78.34	104	91.84	91.25	7.34
SiO_2	2	8	11	9.5	9.5	2.12
pH	12	7.39	8	7.71	7.72	0.2
TDS	12	506	622.5	553.02	546.5	33.82
EC	12	581	699	643.75	653	38.44
Well #10 (ARA)						
Parameter	*N. Obs*	*Min*	*Max*	*Mean*	*Median*	*SD*
HCO_3	12	170	305	266	268	32.9
Cl	12	30.3	60	44.4	42.4	9.9
NO_3	12	6	21	12.7	11.5	5.64
SO_4	12	69.2	97	80.1	79.7	7.17
Na	12	27.7	47	33.8	32.7	4.84
K	12	2.3	3.2	2.85	2.9	0.23
Mg	12	14.5	19	17.3	17.6	1.25
Ca	12	78	114	90.8	88.9	10

SiO_2	2	9	12	10.5	10.5	2.12
pH	12	7	8.21	7.61	7.62	0.3
TDS	12	479	599	548	553	34.9
EC	12	584	721	639	649	40.6
Well #11 (AP - urban area)						
Parameter	*N. Obs*	*Min*	*Max*	*Mean*	*Median*	*SD*
HCO_3	7	390	434	416	416	15.2
Cl	7	90	101	93.7	92.3	3.57
NO_3	7	55	79.7	67.2	64.8	8.84
SO_4	7	97	110	102	100	5.07
Na	7	62.3	73.3	65.8	65	3.7
K	7	4.1	4.8	4.31	4.29	0.23
Mg	7	27.2	32.2	29.4	29	1.94
Ca	7	132	172	154	151	14.49
SiO_2	1	16	16	-	-	-
pH	7	7	7.33	7.2	7.26	0.12
TDS	7	892	955	932	930	21.64
EC	7	1053	1098	1074	1079	17.45
Well #12 (AP - urban area)						
Parameter	*N. Obs*	*Min*	*Max*	*Mean*	*Median*	*SD*
HCO_3	7	384	427	411	415	14.2
Cl	7	119	180	155	153	20.4
NO_3	7	49	58	52.9	52.7	2.86
SO_4	7	54.5	101	80	79.6	13.7
Na	7	81.6	99.3	90.5	92.3	6.59
K	7	3.79	4.1	3.96	4	0.1
Mg	7	29.7	38	32.12	31.4	2.71
Ca	7	105	171	136	131	22.6
SiO_2	2	15	18	16.5	16.5	2.12
pH	7	7	7.41	7.2	7.18	0.14
TDS	7	936	1004	961	953	27.1
EC	7	1134	1269	1184	1185	44.4
Well #13 (AP - industrial-central area)						
Parameter	*N. Obs*	*Min*	*Max*	*Mean*	*Median*	*SD*
HCO_3	2	239	422	331	331	130
Cl	2	84.3	157	121	121	51.4
NO_3	2	4.82	7.6	6.21	6.21	1.97
SO_4	2	96.4	101	98.7	98.7	3.27
Na	2	43.4	91.1	67.3	67.3	33.7
K	2	2.25	3.6	2.92	2.92	0.95
Mg	2	17.1	39.6	28.3	28.3	16
Ca	2	88.7	172	130	130	58.9
SiO_2	1	10	10	-	-	-
pH	2	7.09	7.65	7.37	7.37	0.4
TDS	2	576	994	785	785	296
EC	2	743	1257	1000	1000	363
ell #14 (AP - coastal area)						
Parameter	*N. Obs*	*Min*	*Max*	*Mean*	*Median*	*SD*
HCO_3	4	374	435	405	407	30
Cl	4	93	270	175	168	77.9
NO_3	4	25	135	85.1	90	45.5
NO_2	1	0.42	0.42	-	-	-
SO_4	4	94	156	134	143	28.7
Na	4	63.8	175	109	98.5	47.6

NH_4	1	0.82	0.82	-	-	-
K	4	7.73	10.4	8.98	8.89	1.11
Mg	4	31.3	39	33.9	32.7	3.54
Ca	4	153	178	164	163	10.4
SiO_2	2	13	15	14	14	1.41
pH	4	7.14	7.8	7.45	7.42	0.29
TDS	4	1027	1285	1116	1076	119
EC	4	1248	1540	1389	1384	162
Well #15 (AP - Inland area)						
Parameter	*N. Obs*	*Min*	*Max*	*Mean*	*Median*	*SD*
HCO_3	8	374	425	398	400	20.3
Cl	8	59.4	103	76.6	76.5	15.7
NO_3	8	65	87.8	74.6	73.9	8.55
SO_4	8	70	101	77.6	73.3	10.5
Na	8	45	62.6	51.5	49	6.94
K	8	1.8	3	2.21	2.05	0.41
Mg	8	27.4	36	33.5	34.5	3.03
Ca	8	113	164	131	128	16.3
SiO_2	2	12	15	13.5	13.5	2.12
pH	8	7.11	7.5	7.28	7.28	0.14
TDS	8	785	917	844	845	45.1
EC	8	908	1164	983	949	89
Well #16 (AP - Inland area)						
Parameter	*N. Obs*	*Min*	*Max*	*Mean*	*Median*	*SD*
HCO_3	8	225	424	382	416	71.6
Cl	8	48	62.9	57.5	58	4.78
NO_3	8	13	95	81.6	91.6	27.9
NO_2	4	0.01	0.01	0.01	0.01	0
SO_4	8	86	112.4	106	110	9.81
Na	8	34	49.4	45.9	48	5.07
K	8	3.1	4.39	3.45	3.35	0.4
Mg	8	18	43.4	38.4	42	8.71
Ca	8	84	148	125	135	24.17
SiO_2	1	13	13	-	-	-
pH	8	7.27	7.83	7.49	7.44	0.2
TDS	8	511	926	840	907	145
EC	8	603	1048	953	1011	149
Well #17 (AP - industrial-central area)						
Parameter	*N. Obs*	*Min*	*Max*	*Mean*	*Median*	*SD*
HCO_3	8	217	377	340	355	51.4
Cl	8	52	65.1	60.6	61.1	4.1
NO_3	8	11	100	76.9	83.1	28.2
NO_2	4	0.01	0.01	0.01	0.01	0
SO_4	8	83	106	96.6	98.5	7.77
Na	8	35	45	40.2	40.5	2.96
NH_4	4	0.01	0.01	0.01	0.01	0
K	8	2.4	3.4	2.72	2.63	0.33
Mg	8	18	27.4	23.2	24.8	3.21
Ca	8	86	165	137	144	25.9
SiO_2	2	14	18	16	16	2.83
pH	8	7.1	7.94	7.39	7.3	0.25
TDS	8	511	859	778	814	113
EC	8	613	974	893	927	116
Well #18 (AP - industrial-central area)						

Parameter	*N. Obs*	*Min*	*Max*	*Mean*	*Median*	*SD*
HCO_3	4	417	508	471	479	38.6
Cl	4	52.4	107	74.8	69.9	26.4
NO_3	4	22.5	67.5	40.3	35.5	21.6
SO_4	4	67.5	183	119	113	47.5
Na	4	58.7	86.9	67.8	62.7	13.1
K	4	3.5	4.15	3.78	3.74	0.33
Mg	4	34	41.6	36.7	35.7	3.54
Ca	4	118	146	128	123	12.8
SiO_2	2	16	18	17	17	1.41
pH	4	7.16	7.32	7.22	7.19	0.07
TDS	4	889	994	941	939	47.6
EC	4	973	1320	1112	1078	160
Well #19 (AP - industrial-central area)						
Parameter	*N. Obs*	*Min*	*Max*	*Mean*	*Median*	*SD*
HCO_3	7	354	376	363	361	7.05
Cl	7	61	69.5	65	64.7	3.54
NO_3	7	79	113	94.4	95	13.6
SO_4	7	77.3	96.5	90.1	94.5	8.27
Na	7	37.5	45.8	41.9	42	2.68
K	7	2.22	2.8	2.5	2.46	0.21
Mg	7	18.8	25.3	22.07	22.7	2.37
Ca	7	120	167	144	148	17
SiO_2	2	16	18	17	17	1.41
pH	7	7.17	7.43	7.3	7.3	0.11
TDS	7	753	877	823	831	43.2
EC	7	896	998	953	970	41.7
Well #20 (AP - industrial-central area)						
Parameter	*N. Obs*	*Min*	*Max*	*Mean*	*Median*	*SD*
HCO_3	8	404	425	412	411	6.83
Cl	8	81.8	92.8	88.1	88.3	4.08
NO_3	8	89	119	101	99.7	12.7
SO_4	8	106	123	114	113	5.47
Na	8	59.2	66.4	62.3	62	2.49
K	8	2.84	3.6	3.28	3.3	0.21
Mg	8	35	41.2	37.9	38.4	2
Ca	8	124	160	145	148	13.2
SiO_2	2	16	18	17	17	1.41
pH	8	7	7.53	7.23	7.27	0.16
TDS	8	948	990	963	959	15.2
EC	8	1095	1147	1113	1111	17.3
Well #21 (AP - coastal area)						
Parameter	*N. Obs*	*Min*	*Max*	*Mean*	*Median*	*SD*
HCO_3	10	386	473	430	433	30.3
Cl	10	108	132	119	121	6.71
NO_3	10	51	123	72.5	63.7	24.2
SO_4	10	101	140	121	124	12.8
Na	10	76	120	102	107	14.9
K	6	0.01	0.18	0.04	0.01	0.07
NH_4	10	2.16	4.3	3.63	3.75	0.65
Mg	10	30	35	32.03	31.42	1.65
Ca	10	94.7	157	137	142	16.8
SiO_2	2	9	15	12	12	4.24
pH	10	7.1	7.44	7.31	7.38	0.14
TDS	10	954	1095	1018	1010	42.4

EC	10	1123	1259	1195	1217	54.1

Well #22 (AP - coastal area)						
Parameter	*N. Obs*	*Min*	*Max*	*Mean*	*Median*	*SD*
HCO_3	10	378	573	459	448	55.6
Cl	10	45.9	211	104	86.8	56
NO_3	10	16	180	67.1	58	42.9
SO_4	10	73.9	192	125	112	42.3
Na	10	50.8	228	100	75.1	59.5
NH_4	6	0.01	0.08	0.02	0.01	0.03
K	10	2.7	15	5.88	4.05	3.99
Mg	10	23.4	44.7	32.73	31.7	6.62
Ca	10	99.8	166	136.8	141	23
SiO_2	2	9	12	10.5	10.5	2.12
pH	10	7	7.52	7.32	7.34	0.13
TDS	10	772	1374	1030	989	200
EC	10	865	1685	1175	1118	269

Well #23 (AP - coastal area)						
Parameter	*N. Obs*	*Min*	*Max*	*Mean*	*Median*	*SD*
HCO_3	6	376	515	446	443	58.5
Cl	6	76	233	159	164	58.2
NO_3	6	38.7	130	76.4	67.2	37.5
SO_4	6	115	148	134	136	12.8
Na	6	57	199	125	118	59.9
NH_4	3	0.01	1.45	0.49	0.01	0.83
K	6	3.6	10.4	7.39	7.55	2.57
Mg	6	22.2	32.9	28.6	28.8	4.04
Ca	6	104	158	144	150	19.9
SiO_2	2	11	14	12.5	12.5	2.12
pH	6	7.15	7.64	7.39	7.4	0.2
TDS	6	917	1299	1119	1115	150
EC	6	1034	1704	1356	1350	242

Well #24 (AP - coastal area)						
Parameter	*N. Obs*	*Min*	*Max*	*Mean*	*Median*	*SD*
HCO_3	8	376	465	423	426	32.6
Cl	8	52.7	86.9	70.7	70.1	12.2
NO_3	8	60.7	111	79.3	75.2	19.7
SO_4	8	102	182	126	119	24
Na	8	56	64.7	59.7	58.4	3.25
K	8	8.8	10.7	10	9.9	0.61
Mg	8	26.5	36.3	31.3	31.1	2.88
Ca	8	122	166	152	157	14
SiO_2	2	10	15	12.5	12.5	3.54
pH	8	7.1	8.03	7.38	7.33	0.28
TDS	8	853	1113	953	937	77.5
EC	8	965	1291	1090	1048	114.5

Well #25 (AP - coastal area)						
Parameter	*N. Obs*	*Min*	*Max*	*Mean*	*Median*	*SD*
HCO_3	7	368	471	421	426	30.7
Cl	7	60.6	93	75.1	75.2	12.5
NO_3	7	57.9	109	78.3	68.2	21.1
SO_4	7	103	171	124	118	23
Na	7	58	70.1	63.4	63.4	4.83
NH_4	4	0.01	0.01	0.01	0.01	0

K	7	9.7	12.5	10.4	10	1
Mg	7	29	37.2	31.5	30	2.93
Ca	7	139	171	153	151	12.4
SiO_2	2	9	17	13	13	5.66
pH	7	7.1	7.97	7.42	7.3	0.33
TDS	7	909	1129	957	928	77.5
EC	7	1025	1291	1108	1053	97.6
Well #26 (AP - Inland area)						
Parameter	*N. Obs*	*Min*	*Max*	*Mean*	*Median*	*SD*
HCO_3	4	373	451	397	382	36.7
Cl	4	39.5	64	52.4	53	11.9
NO_3	4	13.4	36.7	21.7	18.3	10.7
NO_2	1	0.01	0.01	-	-	-
SO_4	4	60	91.8	73.9	71.8	14.8
Na	4	24.5	42.4	32.9	32.3	7.54
K	4	2.35	3.6	3.08	3.19	0.58
Mg	4	14	21.1	16.7	15.9	3.09
Ca	4	119	151	129	122	15.1
SiO_2	2	12	12	12	12	0
pH	4	7.27	7.57	7.44	7.47	0.14
TDS	4	651	770	726	742	52
EC	4	718	872	809	823	65
Well #27 (AP - Industrial-central area)						
Parameter	*N. Obs*	*Min*	*Max*	*Mean*	*Median*	*SD*
HCO_3	9	417	437	429	432	6.97
Cl	9	56.1	67.6	62.3	62.4	3.85
NO_3	9	92	112	100	97.9	6.39
SO_4	9	101	118	110	112	4.92
Na	9	43.7	49.3	46.6	46.9	1.91
K	9	3.05	3.6	3.35	3.4	0.16
Mg	9	36.7	44.4	41.6	42.8	2.48
Ca	9	122	159	141	144	13.5
SiO_2	2	15	19	17	17	2.83
pH	9	7	7.91	7.37	7.38	0.27
TDS	9	903	960	934	937	22
EC	9	1032	1085	1056	1056	19

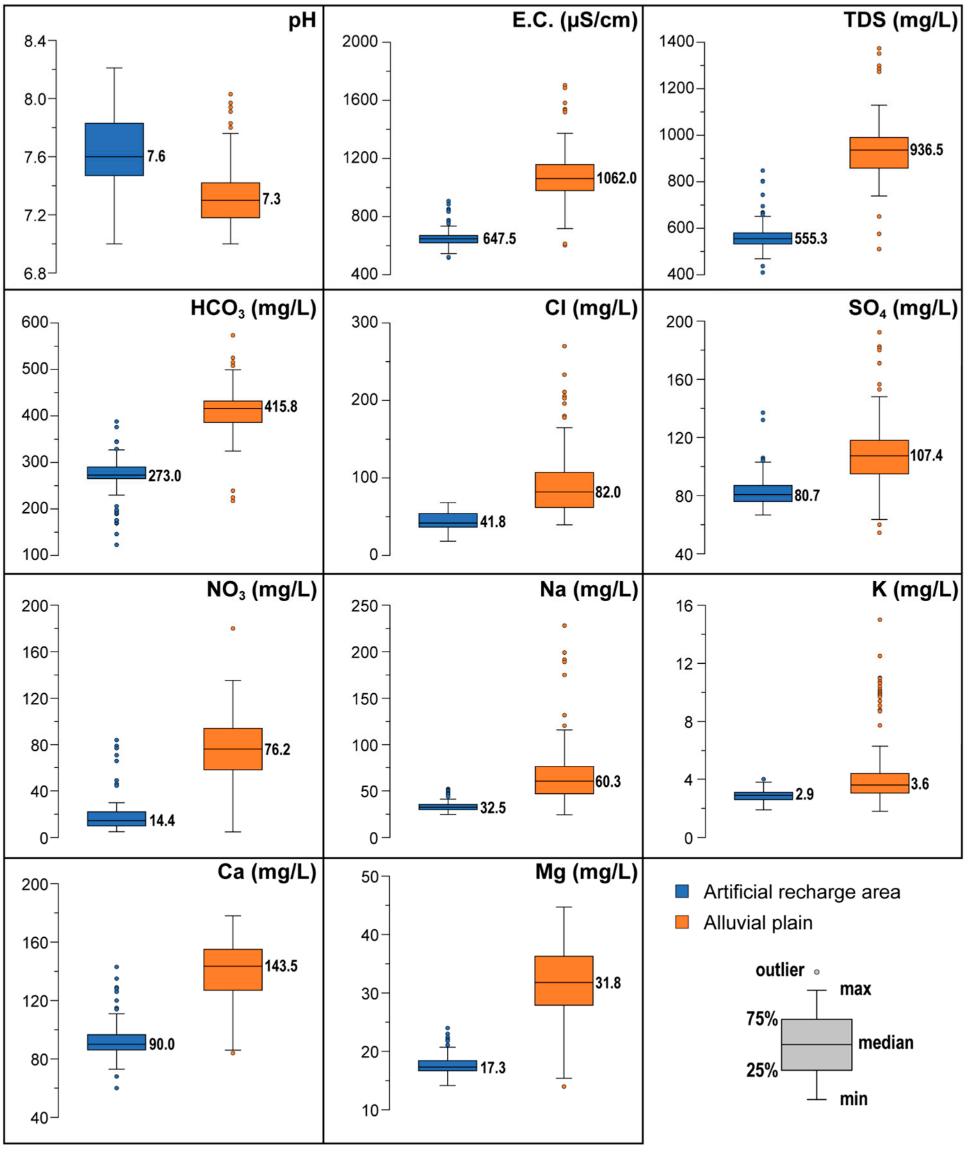

Figure S1. Box-plots of the main physicochemical parameters and main ions divided into Artificial Recharge Area (ARA) and Alluvial Plain (AP) samples, respectively.

Table S2: nitrate content statistical parameters for each investigated well

Nitrates (mg/L)							
Well ID	*Timespan*	*N. Obs*	*Min*	*Max*	*Mean*	*Median*	*SD*
1	2009-2020	92	2	75	22.5	18.4	15.8
2	2009-2020	93	2.7	83.5	22.9	16.1	20
3	2009-2020	94	1	79	26.3	20.3	19.9
4	2009-2020	93	4.8	84	18.6	15	13.1
5	2009-2020	93	4.8	77	18.9	15.4	13.1
6	2009-2020	92	3	65.7	19.3	14.2	15.6
7	2009-2020	93	0.6	89.3	28.8	19.2	24.1
8	2009-2020	93	4	79.2	28.4	20	21.7
9	2009-2020	92	4	57	18.5	12.8	13.9
10	2009-2020	93	4	79.2	15.7	13.7	9.74
11	2009-2020	30	14	97	67.8	67	16.2
12	2009-2019	41	12	92	56.2	54.5	10.6
13	2009-2019	5	4.82	86	53.3	82	43
14	2009-2020	9	25	135	83	86	29.3
15	2009-2020	36	61	104	82.7	84.4	13.5
16	2009-2020	34	10.2	109	90.8	94	21.3
17	2008-2020	17	11	104	80.9	84.2	22
18	2009-2016	26	22.3	97	49.2	49.3	23.8
19	2008-2020	38	19	121	94.8	100	17.5
20	2008-2020	55	20	122	95.1	102	20.9
21	2009-2020	44	15	123	75	68.1	22
22	2009-2020	46	8.7	180	60.4	58.7	32.1
23	2009-2019	30	34.5	130	70.6	70.3	24.4
24	2009-2020	44	16	111	77.7	82.3	15.4
25	2009-2020	43	13	109	77.4	80	16.5
26	2009-2020	36	45	121	101	102	12.9
27	2009-2017	10	13.4	36.7	25	26	8.53
